# Hydrogen Sulfide (H_2_S) Mitigates Sepsis-Induced Adrenal Dysfunction via Inhibition of TNFα-Mediated Necroptosis

**DOI:** 10.3390/pathogens14050439

**Published:** 2025-04-30

**Authors:** Kai Ma, Jingwen Huang, Jin Zhang, Yuan Tian, Jing Hu, Linhao Ma, Changnan Wang

**Affiliations:** 1 Lab of Stress Injury, School of Life Sciences, Shanghai University, Shanghai 200444, China; 13047611672@163.com (K.M.); hjwnsdd@163.com (J.H.); ty990614@163.com (Y.T.);; 2 General Practice Department, Shanghai Pudong New District Kangqiao Community Health Service Center, Shanghai 201315, China; zj_0518@126.com; 3 Department of Emergency Medicine, Shanghai Fourth People’s Hospital, School of Medicine, Tongji University, Shanghai 200081, China

**Keywords:** sepsis, adrenal dysfunction, necroptosis, gene knockout mice, hydrogen sulfide, TNFα

## Abstract

Background: Sepsis is a life-threatening condition that is characterized by systemic inflammation and organ dysfunction, with adrenal dysfunction being a significant complication. This study aimed to investigate the role of necroptosis and hydrogen sulfide (H_2_S) in sepsis-induced adrenal dysfunction. Methods: A cecal ligation and puncture (CLP)-induced sepsis mouse model was employed. Adrenocortical-specific mixed lineage kinase domain-like pseudokinase (MLKL) knockout (MLKL-KO) and cystathioneine β-synthase (CBS) knockout (CBS-KO) mice were generated using Cre-loxP technology and adrenocortical-specific Cre tool mice. In vitro experiments utilized TNFα-stimulated Y1 adrenocortical cells. The treatments included the H_2_S donor NaHS, TNFα inhibitor R-7050, necroptosis inhibitor NSA and CBS inhibitor AOAA. Pathological assessment involved hematoxylin–eosin (H&E) staining and a Western blot analysis of necroptosis markers (the phosphorylation of MLKL (p-MLKL) and phosphorylation of receptor-interacting protein kinases 1 (p-RIPK1)). Results: Sepsis induced adrenal congestion, elevated TNFα levels, and activated necroptosis (increased p-MLKL/p-RIPK1) in wild-type mice. H_2_S treatment attenuated adrenal damage, reduced TNFα, and suppressed necroptosis. MLKL knockout reduced septic adrenal dysfunction, whereas CBS knockout exacerbated septic adrenal dysfunction. In vitro, TNFα induced Y1 cell necroptosis, which was reversed by H_2_S or NSA. AOAA exacerbated TNFα-induced necroptosis in Y1 cells. Conclusions: H_2_S inhibits TNFα-mediated necroptosis, thereby preserving adrenal integrity in sepsis. Targeting the TNFα–necroptosis axis and enhancing endogenous H_2_S production may represent novel therapeutic strategies for sepsis-associated adrenal dysfunction.

## 1. Introduction

Sepsis represents a life-threatening systemic disorder that arises from a dysregulated immune response to infection. This multifaceted condition manifests as critical organ dysfunction due to the interplay of hyperinflammation and immunosuppression, ultimately leading to acute multi-organ failure with significant mortality risk. Notably, sepsis affects a substantial proportion of critically ill patients globally, accounting for approximately 31.5 million annual fatalities [1,2]. Among its complications, adrenal dysfunction emerges as both a frequent and underappreciated pathological consequence. The adrenal glands secrete various hormones (such as glucocorticoids), which play a crucial role in alleviating inflammatory responses and maintaining homeostasis [3,4]. Clinical studies have revealed that even mild forms of adrenal dysfunction are independently associated with increased mortality in septic patients [5,6]. Despite its clinical relevance, the pathophysiological mechanisms underlying post-sepsis adrenal cortical dysfunction remain incompletely understood, thereby generating considerable debate regarding the optimal application of glucocorticoid therapy in clinical practice [7]. Therefore, elucidating the molecular basis of sepsis-induced adrenal dysfunction holds critical importance for refining evidence-based glucocorticoid management strategies and improving patient prognosis [8].

Necroptosis is a regulated form of programmed cell death. This caspase-independent self-destructive pathway is activated when canonical apoptotic pathways become functionally compromised [9]. Morphologically, necroptosis shares characteristic features of necrosis, including cellular swelling, plasma membrane rupture, and the extracellular leakage of cytoplasmic contents. Distinct from uncontrolled necrosis, however, necroptosis operates through highly coordinated molecular signaling networks [10]. The core molecular machinery of necroptosis involves the serine/threonine kinase complex comprising receptor-interacting protein kinases 1 and 3 (RIPK1/RIPK3) and the mixed lineage kinase domain-like pseudokinase (MLKL) [11,12]. Upon encountering extracellular stimuli (e.g., tumor necrosis factor α [TNFα]) or intracellular danger signals (e.g., microbial nucleic acids), RIPK1 undergoes phosphorylation-dependent activation, which subsequently phosphorylates MLKL (p-MLKL). This post-translational modification triggers MLKL’s translocation to the plasma membrane where it oligomerizes to form lipid pore complexes. These membrane-disrupting structures compromise membrane integrity, promote cytosolic content efflux, and ultimately execute necroptotic cell death [10,11]. Emerging evidence from multiple clinical studies indicates that the adrenal gland may represent a primary target organ for necroinflammatory damage [13]. In septic patients, the levels of RIPK3 and MLKL in serum are significantly elevated, with these biomarkers showing strong correlations with the development of multiple organ dysfunction syndrome (MODS) and mortality [14].

Hydrogen sulfide (H_2_S), an endogenous gas signaling molecule, has garnered escalating attention as the third such molecule discovered following nitric oxide and carbon monoxide [15]. In recent years, H_2_S has become a focal point of research. It exhibits a wide spectrum of biological functions and performs a crucial role in numerous physiological and pathological processes [16,17]. H_2_S can inhibit the Na-K-2Cl symporter and Na-K ATPase in the ascending limb of Henle’s loop, potentially increasing the excretion of sodium and potassium in the urine and participating in renal excretory functions [18]. It has been reported that H2S downregulates cAMP by inhibiting adenylyl cyclase activity, thereby regulating renin release and controlling blood pressure [19]. The synthesis of endogenous H_2_S primarily takes place via the enzymatic actions of cystathioneine β-synthase (CBS), cystathioneine γ-lyase (CSE), and 3-mercaptopyruvate thiotransferase (3-MST) [15]. It has been discovered that H_2_S has the potential to alleviate organ damage related to sepsis by modulating autophagy, suppressing inflammatory responses, and mitigating oxidative stress [20,21]. Based on our previous research, it has been demonstrated that endogenous H_2_S plays a vital role in maintaining adrenal mitochondrial function [15]; we also found that both CBS and CSE are present in murine adrenocortical cells and are responsible for H_2_S generation in the adrenal glands [22]. Meanwhile, H_2_S has been shown to inhibit necroptosis [23,24]. Our previous research findings demonstrated that H_2_S can ameliorate sepsis-induced adrenal dysfunction [22]. However, the precise mechanism by which H_2_S regulates sepsis-induced adrenal dysfunction remains elusive and warrants further investigation.

In this study, we observed a significant upregulation of necroptosis in the adrenal tissues of septic mice, which was effectively suppressed by exogenous H_2_S supplementation, thereby alleviating adrenal tissue damage.

## 2. Materials and Methods

### 2.1. Cell Culture

Y1 cells (mouse adrenal cortical cells) derived from the Chinese Academy of Sciences were cultured in a DMEM medium (basal media) supplemented with 5% FBS at a temperature of 37 °C in a 5% CO_2_–95% air environment. The medium was changed every 2 days. Passages 2 to 6 were used in the in vitro experiments.

The Y1 cells were randomly allocated into three groups: control, TNFα, and TNFα + NaHS. The cells were planted in twelve-well plates at a density of 4.5 × 10^5^ cells/well and cultured in the aforementioned media at a temperature of 37 °C in a 5% CO_2_–95% air environment.

### 2.2. Cell Treatment

Y1 cells were treated with TNFα (TargetMol, Shanghai, China) at 100 ng/mL, with NSA (TargetMol, Shanghai, China) at 0.5 μM, AOAA (Selleck, Houston, TX, USA) at 0.5 mM, or NaHS at 1 μM, 10 μM, and 100 μM.

### 2.3. Animals and Experimental Protocols

Adult male C57BL/6 mice, weighing 20–25 g, were purchased from the Shanghai SLAC Laboratory Animal Co. (Shanghai, China). CBS ^f/f^ and MLKL ^f/f^ mice on a C57BL/6J background were originally obtained from the Jackson Laboratory (Bar Harbor, ME, USA). C57BL/6 mice were used in the present study. All animals were housed with regular light–dark cycles (lights on at 7:00 AM; lights off at 7:00 PM) under a controlled temperature (22 ± 2 °C) and humidity (50 ± 10%) and were provided a standard diet and water ad libitum. All animal protocols were approved by the ethics committee of Experimental Animals of Shanghai University.

Our laboratory generated a novel transgenic mouse line expressing Cre recombinase specifically in the adrenal cortex zona fasciculata. By crossing this tool strain with conditional knockout mice, we achieved the targeted gene deletion in the adrenal cortex while maintaining normal CTH expression levels in non-target organs, including the heart, liver, spleen, lung, and kidney [25]. Subsequently, we crossed this Cre-expressing mouse with CBS ^f/f^ and MLKL ^f/f^ conditional knockout strains to generate CBS ^f/f^/Cre (CBS-KO) and MLKL ^f/f^/Cre (MLKL-KO) mice, respectively. Mice were bred in-house, per proper laboratory animal care. The two groups of mice used were 8-week-old male CBS/MLKL-KO mice, with non-Cre-expressing littermates serving as controls. CBS/MLKL-KO mice were generated that lacked CBS/MLKL expression. These mice were fed a standard rodent chow diet (Zelgler Bros Inc., Gardners, PA, USA) with constant water and food access. All animal protocols were approved by the ethics committee of Experimental Animals of Shanghai University.

Adult male mice were randomly divided into the following groups: control, CLP, CLP + NaHS, and CLP + R-7050. NaHS was dissolved in sterile pyrogen-free saline and injected i.p. at a dose of 1 mg/kg or 10 mg/kg at 16:00–16:30 [26,27]. R-7050 (TargetMol, Shanghai, China) was dissolved in sterile saline and injected i.p. immediately before the CLP surgery at a dose of 10 mg/kg. The control group received an equivalent volume of saline at 16:00–16:30.

### 2.4. Cecal Ligation and Puncture Methods

Male 8-week-old mice were housed under controlled room temperature with free access to food and water under a natural day/night cycle. All animal protocols were approved by the ethics committee of Experimental Animals of Shanghai University. Small mice weighing between 20 and 25 g were selected. After administering an appropriate dose of tribromoethyl alcohol (TargetMol, Shanghai, China) based on their respective body weights, the animals were placed in a supine position and immobilized on a surgical board. The abdominal fur was gently removed using a depilatory agent, and the area was sterilized using a cotton ball soaked in 75% alcohol. Sterile surgical instruments were used to make an approximately 1 cm incision in the lower abdomen of the animals. The cecum was located, and we used a 5.0 silk suture (Teleflex, Shanghai, China) to ligate the outer third portion and puncture twice (with a 21-gauge needle) without passing through to the other side. After ligation, a needle was inserted into the cecum from the mesentery side to the anti-mesentery side, and a drop of feces was extruded from the puncture hole. The cecum was then returned to the abdominal cavity, and the peritoneum and skin were sutured. The mice were euthanized 24 h later; then, adrenal tissues were collected for experiments. The 24 h survival rate of CLP mice was between 60% and 70%.

### 2.5. Inflammation Detection

The inflammation factor experiment was mainly conducted by Xitang Biological Company (Shanghai, China) using the double-antibody sandwich ABC-ELISA method. The samples were serum. The specific procedures were as follows:

Prepare the standard solution: Take 8 × 1.5 mL centrifuge tubes; add 900 μL of specimen diluent to the first tube and 500 μL of specimen diluent to the second through eighth tubes. Then, add 100 μL of 5 ng/mL standard solution to the first tube, mix thoroughly on a vortex mixer, and pipette 500 μL into the second tube. Repeat this serial dilution process. Discard 500 μL from the seventh tube. The eighth tube serves as the blank control.

Testing Procedure: Add 100 μL of the standard solution or test sample to each well. Mix the reaction plate thoroughly and incubate at 37 °C for 40 min. Wash the reaction plate thoroughly 4–6 times with washing buffer and blot dry on filter paper. Add 50 μL of distilled water and 50 μL of the first antibody working solution to each well. Mix the reaction plate thoroughly and incubate at 37 °C for 20 min. Wash the plate again and add 100 μL of enzyme-labeled antibody working solution to each well. Incubate the reaction plate at 37 °C for 10 min before washing the plate again. Add 100 μL of substrate working solution to each well and incubate at 37 °C in the dark for 15 min. Add 100 μL of stop solution to each well and mix thoroughly. Finally, measure the absorbance within 30 min using an ELISA reader (ThermoFisher, Waltham, MA, USA) at 450 nm.

### 2.6. Western Blot Analysis

Adrenal tissues and Y1 cells were homogenized in cold T-Per lysis buffer (Pierce Biotechnology, Inc., Waltham, MA, USA). Approximately 30 mg/lane of protein samples of tissues, cells, mitochondria, and cytosolic fraction were separated using a 10% SDS PAGE gel and subsequently transferred to nitrocellulose membranes. After blockage in 5% skim milk powder in 0.1% Tris-buffered saline/Tween 20 (TBST) for 2 h, the samples were incubated with antibodies against MLKL (Proteintech, Wuhan, China), P-MLKL (Proteintech, Wuhan, China), RIPK1 (Proteintech, Wuhan, China), P-RIPK1 (Proteintech, Wuhan, China), or β-Actin (Proteintech, Wuhan, China) overnight at 4 C at a dilution of 1:500 or 1:1000. Then, the membranes were incubated with a secondary horseradish peroxidase-conjugated antibody (Proteintech, Wuhan, China) for 1 h at room temperature. Immunoreactive proteins were visualized using an enhanced chemiluminescent Western blotting detection system (Santa Cruz Biotechnology, Santa Cruz, CA, USA). The band intensities were quantified using ImageJ software (Version 1.8.0.112).

### 2.7. Immunofluorescence Staining

Adrenal tissue sections (5–7 μm thick) were first fixed in 4% paraformaldehyde (ThermoFisher, Waltham, MA, USA) at room temperature for 2 h, followed by permeabilization with 0.1% Triton X-100 (15 min). After blocking with 5% bovine serum albumin (BSA) in PBS for 1 h, sections were incubated with primary antibodies (anti-P-MLKL, 1:200) at 4 °C overnight. Subsequently, sections were washed thrice in PBS and incubated with secondary antibodies conjugated to Alexa Fluor 488/594 (1:500, ThermoFisher, Waltham, MA, USA) for 1 h at room temperature. Nuclei were stained with 4′,6-diamidino-2-phenylindole (DAPI, 1 μg/mL) for 10 min. After thorough washing, slides were mounted with ProLong Gold antifade reagent (ThermoFisher, Waltham, MA, USA) and analyzed using a confocal microscope (Zeiss LSM 880, Leica, Germany) with a 40× objective. Negative controls and positive controls (which comprised known positive tissue samples) were included to verify specificity.

### 2.8. H&E Staining

Adrenal tissues were fixed in 4% paraformaldehyde (PBS-buffered) at room temperature for 2 h, followed by sequential dehydration through 70–90–100% ethanol (10 min each) and xylene clearing (5 min twice). Paraffin-embedded sections (5–7 μm thick) were prepared using a microtome. For staining, sections were incubated in Mayer’s hematoxylin solution (1 min) and differentiated in 1% HCl ethanol until nuclei appeared uniformly blue (30 s). Subsequently, sections were stained with eosin Y solution (1 min) and dehydrated again through 70–100% ethanol. After clearing with xylene, slides were mounted with neutral-buffered formalin (NBF)-based medium and covered with glass slides. Histopathological evaluation was performed using an Axio Observer Z3 microscope (Carl Zeiss) with a 20× objective. Negative controls (comprising non-tissue samples) and positive controls were included to ensure staining specificity.

### 2.9. Cell Counting Kit-8 (CCK-8)

Cell viability was assessed using the CCK-8 Cell Proliferation Assay Kit (MERCK, Darmstadt, Germany). Cells were seeded into a 96-well plate at a density of 5000 cells/well. The plate was incubated in a cell culture incubator at 37 °C, 5% CO_2_, and 95% air humidity overnight to allow for cell attachment. Based on experimental design, different concentrations of the drug(s) were added to the wells. Untreated controls were included in the wells to serve as a reference for subsequent comparisons of cell proliferation and viability. The CCK-8 reagent was removed from the refrigerator and allowed to equilibrate to room temperature. The reagent was thoroughly mixed. Then, 10–20 µL of CCK-8 reagent was added to each well, avoiding bubble formation. The plate was returned to the incubator and continued culturing for 1–4 h. The incubation time was adjusted based on cell type and experimental requirements to ensure a complete reaction between CCK-8 and cells. After incubation, the plate was removed from the incubator and gently shaken to distribute the cells uniformly. The absorbance of each well was measured using a microplate reader at a wavelength of 450 nm. A blank control was included in the well (containing only medium and CCK-8 reagent) to subtract background absorbance. The absorbance data were analyzed to evaluate cell proliferation and viability.

### 2.10. Genetic Identification

Samples were collected from 9- to 14-day-old mice, during which time the tail length is optimal for minimally invasive tissue collection. Tissues were either immediately processed or stored at −20 °C for future use. Genomic DNA was isolated using a commercial kit (SparkJade, Shanghai, China). Tissue lysates were incubated with lysis buffer at 55 °C for 30 min, followed by Proteinase K addition and incubation at 95 °C for 5 min. Samples were then centrifuged to clarify supernatants. For each sample, Taq Enzyme Mix, DNA template (supernatant), and gene-specific primers were added. The reaction volume was adjusted with ddH_2_O. Primer pairs were optimized for distinct target genes using qPCR thermal cycling parameters (e.g., annealing temperature). Agarose gels (2–3%) were prepared by heating with TAE buffer and SYBR Green. After gel solidification, PCR products, negative controls, wild-type controls, and DNA ladders were loaded. Electrophoresis was conducted at 120 V for 20–30 min (depending on fragment size) using 1× TAE running buffer. Gel images were visualized under UV light. Genotyping was determined by analyzing band patterns, i.e., wild-type alleles exhibited specific fragment sizes, while mutant phenotypes showed alternative band profiles. A DNA ladder served as a molecular weight standard.

### 2.11. Statistical Analysis

All cell experiments were performed thrice independently, and all the animal experiments were performed six times independently. Data are expressed as the means ± SEMs. The significance of the difference in the mean values among more than two groups was evaluated by one-way analysis of variance (ANOVA), followed by post hoc analysis using the Student–Newman–Keuls test or Bennett’s test, where appropriate. In the statistical analysis of the CCK8 results, we used the non-parametric analysis method. All statistical analyses were performed with SPSS 16.0 (SPSS, Inc., Chicago, IL, USA). A *p*-value of less than 0.05 was considered statistically significant.

## 3. Results

### 3.1. Septic Adrenal Dysfunction and Necroptosis Upregulation in Mice

**Figure 1:**
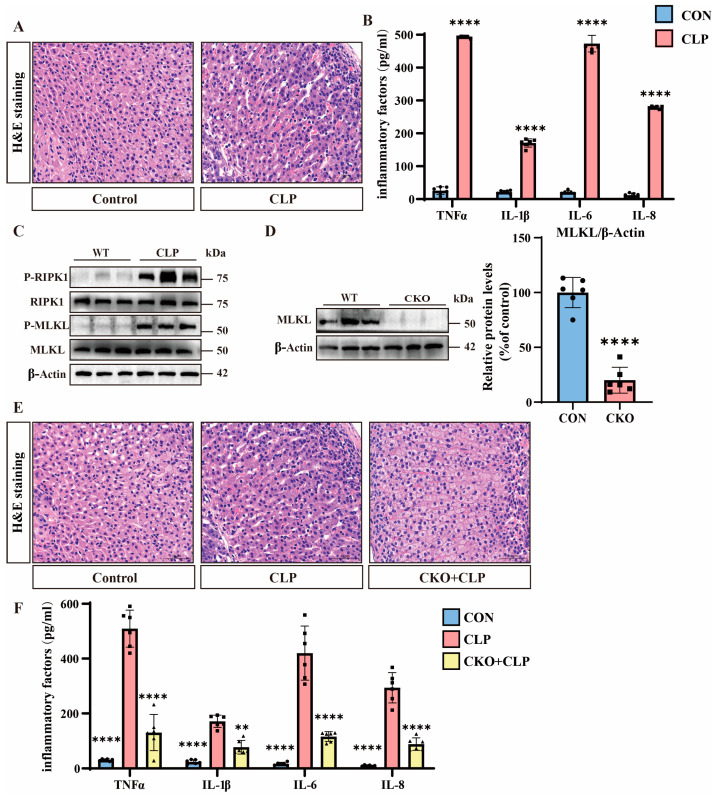
The role of necroptosis in sepsis mice. (**A**) Hematoxylin and eosin (H&E) staining of tissue sections from the control and cecal ligation and puncture (CLP) groups. Scale bar: 50 μm. (**B**) Inflammatory factor expression levels in the control and CLP groups. Data are presented as mean ± standard deviation (SD). **** *p* < 0.0001 indicates statistically significant differences compared to the control group (n = 6 in each group). (**C**) Western blot analysis of RIPK1, P-RIPK1, MLKL, P-MLKL, and β-Actin in the wild-type (WT) and CLP groups (n = 6 in each group). (**D**) Western blot analysis of MLKL in the WT and conditional knockout (MLKL-KO) groups. Data are presented as mean ± standard deviation (SD). **** *p* < 0.0001 indicates statistically significant differences compared to the control group. (**E**) H&E staining of tissue sections from the control, CLP, and MLKL-KO + CLP groups. Scale bar: 50 μm. (**F**) Inflammatory factor expression levels in the control, CLP, and MLKL-KO + CLP groups. Data are presented as mean ± standard deviation (SD). ** *p* < 0.01 and **** *p* < 0.0001 indicate statistically significant differences compared to the CLP group (n = 6 in each group).

We used cecal ligation and puncture (CLP) to construct a septic mouse model. Using hematoxylin–eosin (H&E) staining, we compared adrenal tissues between septic and control mice. Septic mice exhibited marked adrenal congestion and widening intercellular spaces, indicating exacerbated adrenal dysfunction in septic mice (Figure 1A). Meanwhile, the expression of inflammatory factors in the adrenal tissues of septic mice was significantly upregulated (Figure 1B). Emerging evidence has implicated necroptosis in the pathogenesis of sepsis-associated adrenal dysfunction, prompting us to investigate the expression of necroptosis hallmark proteins [28]. Subsequently, we performed Western blot analysis to assess necroptotic pathways. While total MLKL and RIPK1 protein levels remained unchanged, p-MLKL and p-RIPK1 were markedly upregulated in septic adrenal tissues, demonstrating enhanced necroptotic activity (Figure 1C and Figure S1A).

To investigate the functional role of necroptosis, we generated adrenal-specific MLKL knockout mice (MLKL-KO) using Cre-loxP technology and adrenocortical-specific Cre tool mice, with non-Cre-expressing littermates serving as controls [25]. We performed genetic identification using the tails of MLKL-KO mice (Figure S1B). MLKL-KO mice protein analysis confirms a significant reduction in MLKL expression in the adrenal tissues of MLKL-KO mice (Figure 1D). We then constructed a batch of sepsis models using MLKL-KO mice. The H&E staining results show that compared to the adrenal tissues of septic mice, the adrenal tissues of MLKL-KO septic mice exhibited significantly reduced congestion and smaller intercellular spaces, indicating decreased adrenal tissue damage in MLKL-KO septic mice (Figure 1E). Meanwhile, the expression of inflammatory factors in the adrenal tissues of MLKL-KO septic mice was significantly downregulated (Figure 1F).

### 3.2. TNFα-Induced Necroptosis in Y1 Cells and Enhanced Cell Mortality

**Figure 2:**
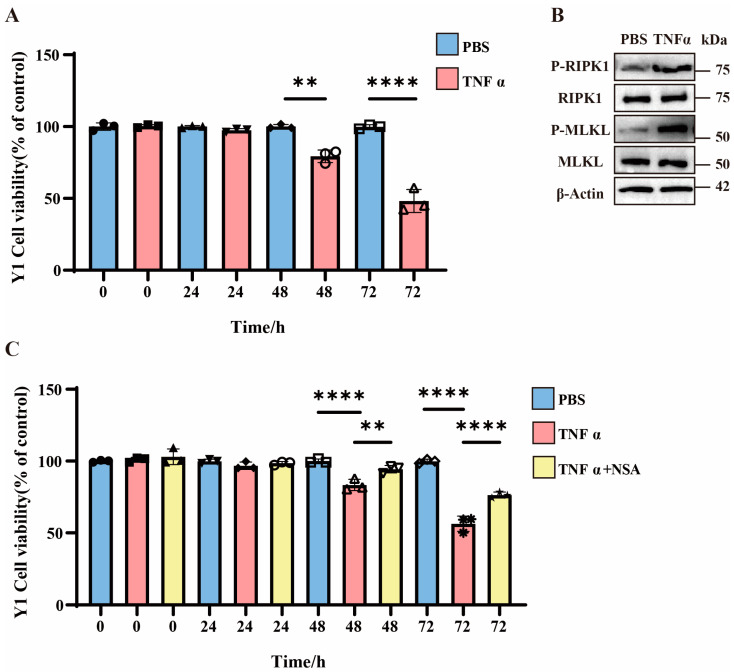
The role of necroptosis in Y1 cells. (**A**) Bar graph showing the viability of Y1 cells treated with PBS or TNFα over time (0, 24, 48, and 72 h). Data are presented as percentage of control (untreated cells). ** *p* < 0.01 and **** *p* < 0.0001 indicate statistically significant differences compared to the control group (n = 3 in each group). (**B**) Western blot analysis of P-RIPK1, total RIPK1, P-MLKL, total MLKL, and β-Actin in Y1 cells treated with PBS or TNFα for 48 h (n = 3 in each group). (**C**) Bar graph showing the viability of Y1 cells treated with PBS, TNFα, or TNFα plus NSA over time (0, 24, 48, and 72 h). Data are presented as percentage of control (untreated cells). ** *p* < 0.01 and **** *p* < 0.0001 indicate statistically significant differences compared to the control group (n = 3 in each group).

To investigate necroptotic mechanisms in vitro, we employed Y1 cells treated with TNFα, which is a well-characterized necroptosis inducer [29,30]. The CCK8 assays reveal that TNFα treatment progressively increased Y1 cell mortality, with significant elevation observed at 48 h and further augmentation at 72 h compared to PBS controls (Figure 2A). Western blot analysis demonstrates no changes in total MLKL or RIPK1 protein levels but a marked upregulation of P-MLKL and P-RIPK1 in TNFα-treated cells, indicating the activation of necroptotic pathways (Figure 2B and Figure S1C). Subsequently, we evaluated the impact of necroptosis inhibition using NSA. The CCK8 data show that 48 h after TNFα treatment, NSA significantly rescued the mortality rate of Y1 cells, and the protective effect of NSA became more pronounced 72 h later (Figure 2C).

### 3.3. H_2_S Suppresses Necroptosis and Alleviates Septic Adrenal Dysfunction

Our prior studies established that CBS and CES are primary enzymes for endogenous H_2_S synthesis in adrenal tissues and play critical roles in maintaining adrenal function [22].

**Figure 3:**
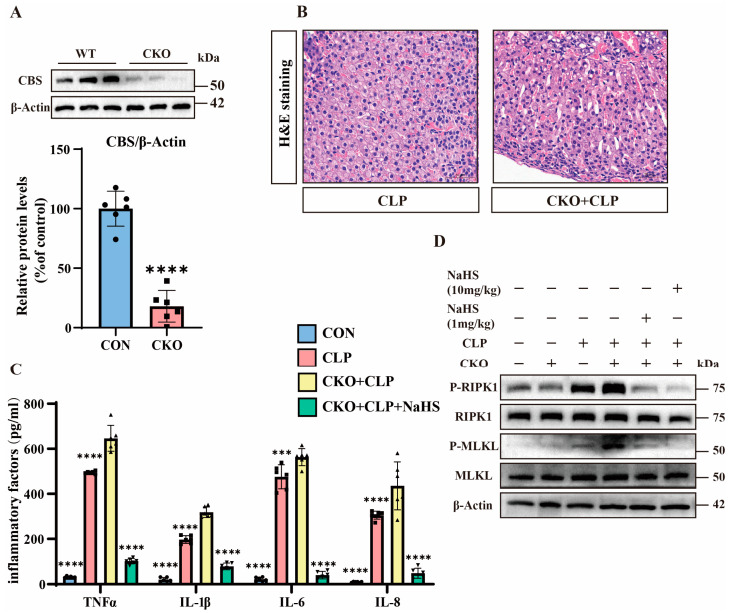
The important role of H_2_S. (**A**) Western blot analysis of CBS and β-Actin protein levels in wild-type (WT) and CBS-KO samples. Data are presented as percentage of control (WT mice). **** *p* < 0.0001 indicates statistically significant differences compared to the control (n = 6 in each group). (**B**) H&E staining of tissue sections from the CLP and CKO + CLP groups. Scale bar: 50 μm. (**C**) Inflammatory factor expression levels in the control, CLP, CBS-KO + CLP, and CBS-KO + CLP + NsHS (10 mg/kg) groups. Data are presented as mean ± standard deviation (SD). *** *p* < 0.001 and **** *p* < 0.0001 indicate statistically significant differences compared to the CBS-KO + CLP group (n = 6 in each group). (**D**) Western blot showing the protein expression levels of P-RIPK1, RIPK1, P-MLKL, MLKL, and β-Actin in the WT, CBS-KO, CLP, CBS-KO + CLP, and CBS-KO + CLP + NaHS (1 mg/kg, 10 mg/kg) groups (n = 6 in each group).

To explore the functional role of H_2_S, we generated CBS adrenal-specific knockout mice (CBS-KO) using Cre-loxP technology and adrenocortical-specific Cre tool mice, with non-Cre-expressing littermates serving as controls [25]. We performed genetic identification using the tails of CBS-KO mice (Figure S2A). Protein analysis demonstrates the near-complete loss of CBS expression in the adrenal tissues of CBS-KO mice (Figure 3A). Pathological evaluation via H&E staining shows that the adrenal tissues of CBS-KO septic mice exhibited aggravated adrenal congestion compared to their control counterparts (Figure 3B). Meanwhile, the expression of inflammatory factors in the adrenal tissues of CBS-KO septic mice was significantly upregulated in comparison to the adrenal tissues of septic mice (Figure 3C). Western blot analysis reveals elevated p-MLKL/p-RIPK1 levels in CBS-KO septic mice compared to septic mice, which were suppressed by NaHS (Figure 3D and Figure S2B). These findings collectively establish that H_2_S can significantly inhibit necroptosis in the adrenal tissues of septic mice.

### 3.4. H_2_S Attenuates TNFα-Induced Y1 Cell Necroptosis Through CBS Activation

**Figure 4:**
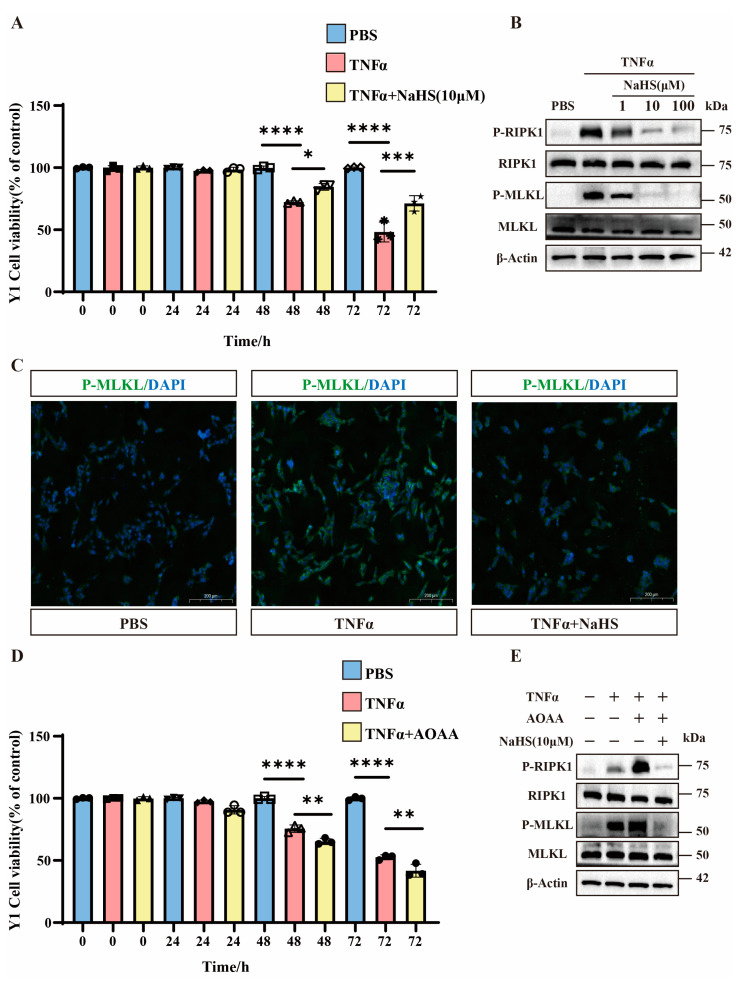
The important role of H_2_S in Y1 cells. (**A**) Bar graphs showing the cell viability of Y1 cells treated with PBS, TNFα, and TNFα combined with NaHS (10 μM) over different time periods (0, 24, 48, and 72 h). Data are presented as percentage of control (untreated cells). * *p* < 0.05, *** *p* < 0.001, and **** *p* < 0.0001 indicate statistically significant differences compared to the control (n = 3 in each group). (**B**) Western blot analysis of protein expression levels of P-RIPK1, RIPK1, P-MLKL, MLKL, and β-Actin in Y1 cells treated with TNFα and NaHS (1 μM, 10 μM, and 100 μM). The blots show the changes in protein expression under different treatment conditions (n = 3 in each group). (**C**) Immunofluorescence images showing the localization of P-MLKL in Y1 cells treated with PBS, TNFα, and TNFα combined with NaHS. The images demonstrate the effect of these treatments on P-MLKL distribution within the cells. Scale bar: 200 μm. (**D**) Bar graphs show the cell viability of Y1 cells treated with PBS, TNFα, and TNFα combined with AOAA over different time periods (0, 24, 48, and 72 h). Data are presented as percentage of control (untreated cells). ** *p* < 0.01 and **** *p* < 0.0001 indicate statistically significant differences compared to the control (n = 3 in each group). (**E**) Western blot analysis of protein expression levels of P-RIPK1, RIPK1, P-MLKL, MLKL, and β-Actin in Y1 cells treated with TNFα, AOAA, and NaHS (10 μM). The blots show the changes in protein expression under different treatment conditions (n = 3 in each group).

The CCK8 assays demonstrate that 48 h after TNFα treatment, H_2_S treatment significantly reduced TNFα-induced Y1 cell mortality. Additionally, 72 h after TNFα treatment, the therapeutic effect of H_2_S became more pronounced (Figure 4A). Western blot analysis confirms that the total MLKL and RIPK1 protein levels remained unchanged, and H_2_S downregulated p-MLKL and p-RIPK1 levels (Figure 4B and Figure S3A). Immunofluorescence staining confirms these findings (Figure 4C).

To investigate the involvement of H_2_S, we used the CBS inhibitor AOAA. The CCK8 data reveal that AOAA significantly exacerbated TNFα-induced cell death at 48 h and further increased mortality at 72 h (Figure 4D). Western blot analysis shows that AOAA potentiated necroptosis (elevated p-MLKL/p-RIPK1) compared to TNFα alone, while NaHS treatment reversed this effect (Figure 4E and Figure S3B).

### 3.5. H_2_S Modulates TNFα-Mediated Inflammation and Necroptosis in Septic Mice

**Figure 5:**
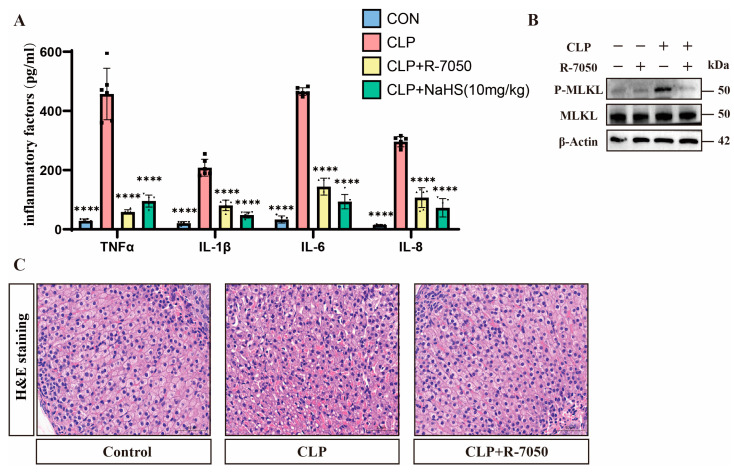
TNFα plays an important role in H_2_S-inhibited necroptosis. (**A**) Inflammatory factor expression levels in the control, CLP, CLP + R-7050, and CLP + NsHS (10 mg/kg) groups. Data are presented as mean ± standard deviations (SDs). **** *p* < 0.0001 indicates statistically significant differences compared to the CLP group (n = 6 in each group). (**B**) Western blot analysis demonstrating the expression levels of P-MLKL and MLKL in the control, CLP, and CLP + R-7050 groups. β-Actin was used as a loading control (n = 6 in each group). (**C**) H&E staining of tissue sections from the control, CLP, and CLP + R-7050 groups, showing the histological changes in response to CLP and R-7050 treatment. Scale bar: 50 μm.

Analyzing the inflammatory markers showed that septic mice exhibited significantly elevated serum TNFα levels, which were reduced by H_2_S treatment (Figure 5A). Western blot analysis demonstrates that the TNFα inhibitor R-7050 reduced the p-MLKL expression of adrenal tissues compared to that of the adrenal tissues of septic mice without affecting total MLKL levels (Figure 5B and Figure S2C). Pathological evaluation via H&E staining shows that the adrenal tissues of septic mice treated with R-7050 exhibited significantly reduced congestion and smaller intercellular spaces compared to the adrenal tissues of septic mice (Figure 5C). Meanwhile, the expression of inflammatory factors in the adrenal tissues of septic mice treated with R-7050 was significantly downregulated in comparison with septic mice (Figure 5A).

## 4. Discussion

In this study, we elucidated the protective role of H_2_S in sepsis-induced adrenal dysfunction through the inhibition of TNFα-mediated necroptosis. Utilizing CLP-induced septic mice and TNFα-stimulated Y1 adrenocortical cells, we demonstrated that H_2_S attenuated adrenal dysfunction, reduced TNFα levels, and suppressed necroptotic signaling (p-MLKL/p-RIPK1).

Necroptosis, an inflammatory form of programmed cell death, contributes significantly to sepsis pathogenesis [14]. Previous studies have documented that necroptosis plays a critical pathological role in both septic lung and liver damage [31,32]. Through the release of damage-associated molecular patterns (DAMPs), such as S100A9, necroptotic cells induce necrotic inflammation. Under the pathophysiological context of pulmonary arterial hypertension (PAH), necroptosis-induced cell loss has been implicated in mediating organ dysfunction [33]. Myocardial dysfunction caused by sepsis (SIMD) is an important cause of death in patients with sepsis. Necroptosis is closely related to SIMD and may lead to myocardial dysfunction through inflammatory responses and cell death [34]. Furthermore, recent studies have identified phosphorylated MLKL (p-MLKL) expression in the septic adrenal tissues of COVID-19 patients, suggesting a potential pathological link between necroptosis and adrenal dysfunction [35]. However, direct evidence linking necroptosis activation to adrenal dysfunction remains limited. Our work establishes that necroptosis is a major driver of septic adrenal dysfunction, as evidenced by its upregulation in CLP-induced sepsis mice and the attenuation of tissue damage following necroptosis inhibition. Furthermore, we generated the first adrenocortical MLKL-specific knockout mouse model and revealed that MLKL knockdown significantly ameliorates sepsis-induced adrenal dysfunction in mice, suggesting that necroptosis is the primary mechanism of adrenocortical cell death during sepsis.

Endogenous H_2_S plays a critical role in maintaining adrenal function during sepsis. Previous studies from our lab using lipopolysaccharide (LPS)-induced sepsis models revealed that LPS suppresses the expression of CBS/CSE, which are key enzymes for H_2_S synthesis, leading to adrenal dysfunction. Conversely, the H_2_S donor GYY4137 prevented LPS-induced mitochondria-mediated apoptosis in adrenocortical cells [15,22]. Similar protective effects of H_2_S were observed in a urinary sepsis-induced renal injury model [36,37]. However, the precise molecular mechanisms through which H_2_S alleviates functional impairments in the adrenal glands and other organs remain incompletely elucidated. Our current work demonstrates that H_2_S alleviates septic adrenal dysfunction and improves adrenal function through inhibiting necroptosis. Additionally, we generated CBS-adrenocortical-specific knockout mice. Our data demonstrate that CBS knockdown significantly exacerbates the necroptosis of adrenal tissues and sepsis-induced adrenal dysfunction in mice, whereas exogenous H_2_S administration effectively reverses these pathological outcomes. These findings collectively establish that H_2_S exerts critical regulatory roles in suppressing necroptotic pathways and alleviating septic adrenal dysfunction through its molecular modulatory mechanisms.

Previous studies have shown that TNFα binds to TNFR1 to activate the TRADD-RIPK1 complex. RIPK1 activation recruits RIPK3 and MLKL, leading to MLKL phosphorylation, plasma membrane pore formation, and subsequent necroptosis [38,39,40]. H_2_S is considered to have anti-inflammatory effects and can inhibit inflammatory responses through multiple pathways [41,42]. Studies have shown that H_2_S can reduce the expression of TNF-α, thereby alleviating inflammatory damage [43,44,45]. However, it remains unclear whether hydrogen sulfide inhibits necroptosis by reducing TNFα levels in the adrenal tissues of septic mice. Our study reveals that sepsis induces a marked elevation of TNFα levels, which are subsequently eliminated by H_2_S treatment to suppress necroptotic cell death in adrenal tissues. This process is accompanied by the amelioration of adrenocortical dysfunction. Furthermore, using the TNFα inhibitor R-7050, we confirm that the H_2_S-mediated alleviation of septic adrenal dysfunction is dependent on TNFα elimination. These findings collectively establish that H_2_S primarily exerts its protective effects by reducing TNFα levels to suppress necroptosis, thereby ameliorating sepsis-induced adrenal dysfunction.

## 5. Conclusions

Our work identifies necroptosis as the primary mechanism underlying septic adrenal dysfunction. By inhibiting TNFα production and subsequently blocking RIPK1-RIPK3-MLKL signaling, H_2_S effectively reduces necroptotic cell death, thereby protecting adrenal tissue integrity. These findings highlight the therapeutic potential of targeting the TNFα–necroptosis axis and endogenous H_2_S pathways in septic patients with adrenal compromise.

There are still some unresolved challenges in the current research. For example, the typical pathway of necroptosis depends on RIPK1, RIPK3, and MLKL. After cytokines, such as tumor necrosis factor, bind to death receptors, RIPK1 is activated. RIPK1 recruits and activates RIPK3, and RIPK1 and RIPK3 form a complex called the necrosome. The necrosome phosphorylates MLKL, and the phosphorylated MLKL oligomerizes and translocates to the cell membrane, disrupting the integrity of the cell membrane and ultimately leading to cell death [12]. However, we did not find the phosphorylation of RIPK3 in the model group, suggesting that there may be a non-canonical RIPK1-activated MLKL pathway, but we did not discuss this in this article. Secondly, how H_2_S affects the level of TNFα and further regulates necroptosis remains unclear. These issues will be the focus of our future research.

## Data Availability

The authors declare that the source data are provided in the original paper.

## Supplementary Materials

The following supporting information can be downloaded at: https://www.mdpi.com/article/10.3390/pathogens14050439/s1.
