# Supplementary material for: Hydrogen Sulfide (H2S) Mitigates Sepsis-Induced Adrenal Dysfunction via Inhibition of TNFα-Mediated Necroptosis
Source: Pathogens. 2025 Apr 30;14(5):439. doi: 10.3390/pathogens14050439 (PMC12113818; doi:10.3390/pathogens14050439)
Supplement: Supplementary file 1 [file pathogens-14-00439-s001.zip › pathogens-3557359-supplementary.pdf]

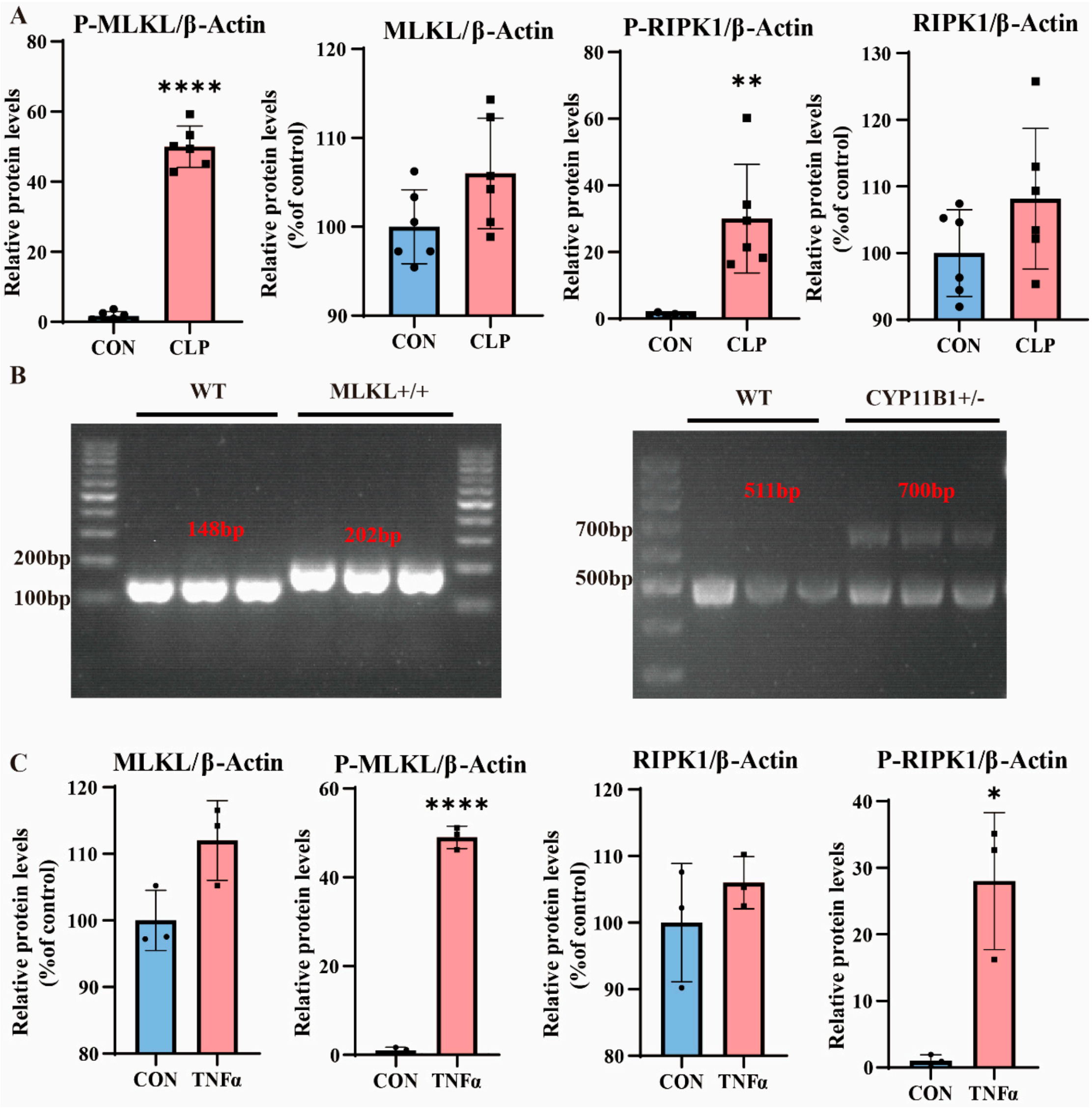

Figure Supplement 1. (A). The statistical plots of Western blot analysis for RIPK1, P-RIPK1, MLKL, P-MLKL, and β-actin in wild-type (WT) and CLP groups of mice. Data are presented as mean ± standard deviation (SD). ****P < 0.0001 indicate statistically significant differences compared to control group. **(B).** The genetic identification results of MLKL-KO and WT mice. The MLKL band was sized at 202 bp, and the CYP11B1 band was sized at 700 bp. (n = 6 in each group) **(C).** The statistical plots of Western blot analysis for RIPK1, P-RIPK1, MLKL, P-MLKL, and β-actin in PBS and TNFα groups of Y1 cells. Data are presented as mean ± standard deviation (SD). ****P < 0.0001 and *P < 0.05 indicate statistically significant differences compared to control group. (n = 3 in each group)

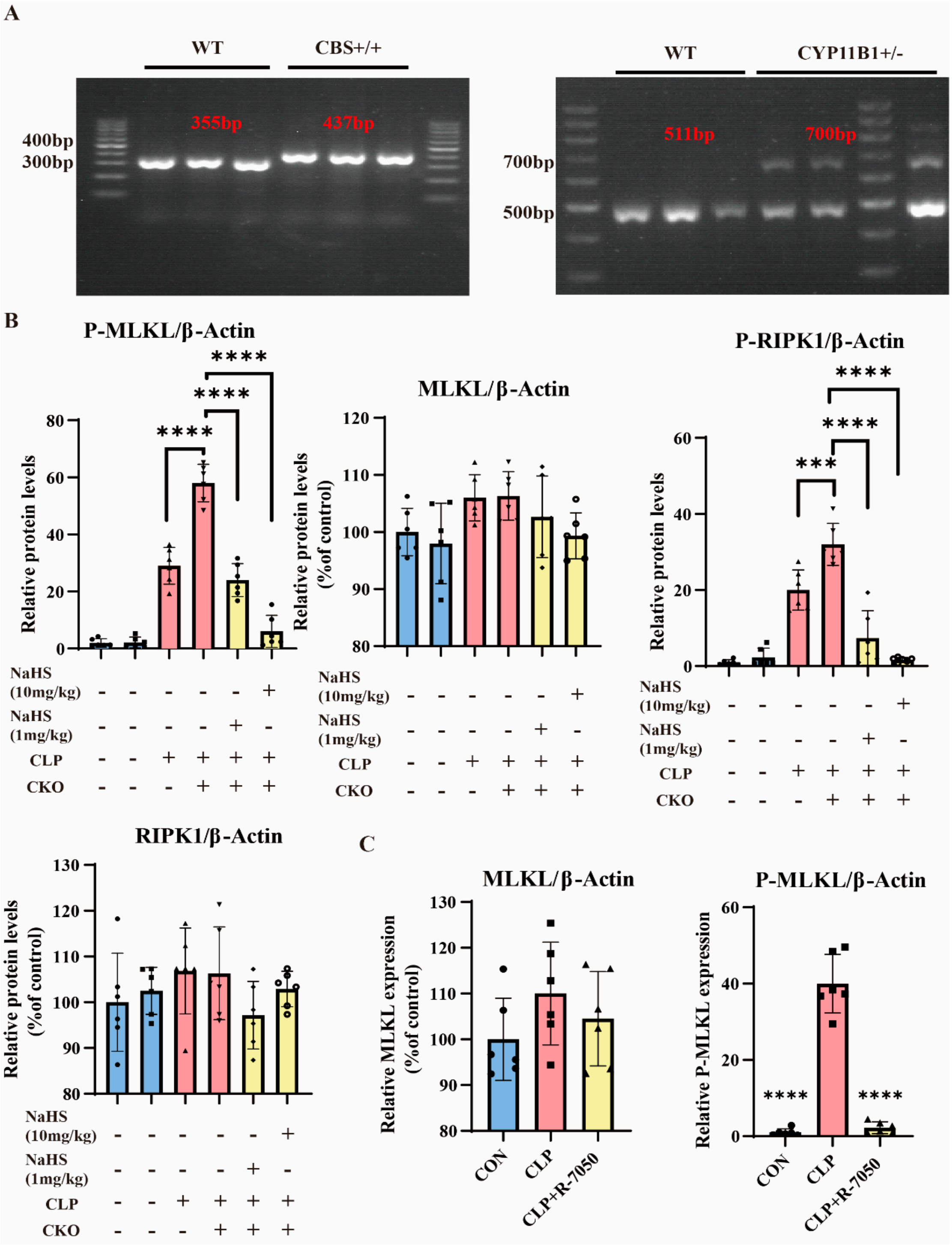

Figure Supplement 2. (A). The genetic identification results of CBS-KO and WT mice. The CBS band was sized at 437 bp, and the CYP11B1 band was sized at 700 bp. **(B).** The statistical plots of Western blot analysis for RIPK1, P-RIPK1, MLKL, P-MLKL, and β-actin in control, CBS-KO, CLP, CBS-KO+CLP and CBS-KO+CLP+NaHS (1mg/kg, 10mg/kg) groups of mice. Data are presented as mean ± standard deviation (SD). ****$P < 0.0001$ and ***$P < 0.001$ indicate statistically significant differences compared to CBS-KO+CLP group. (n = 6 in each group) **(C).** The statistical plots of Western blot analysis for MLKL, P-MLKL, and β-actin in control, CLP and CLP+R-7050 groups of Y1 cells. Data are presented as mean ± standard deviation (SD). ****$P < 0.0001$ indicates statistically significant differences compared to TNFα group. (n = 6 in each group)

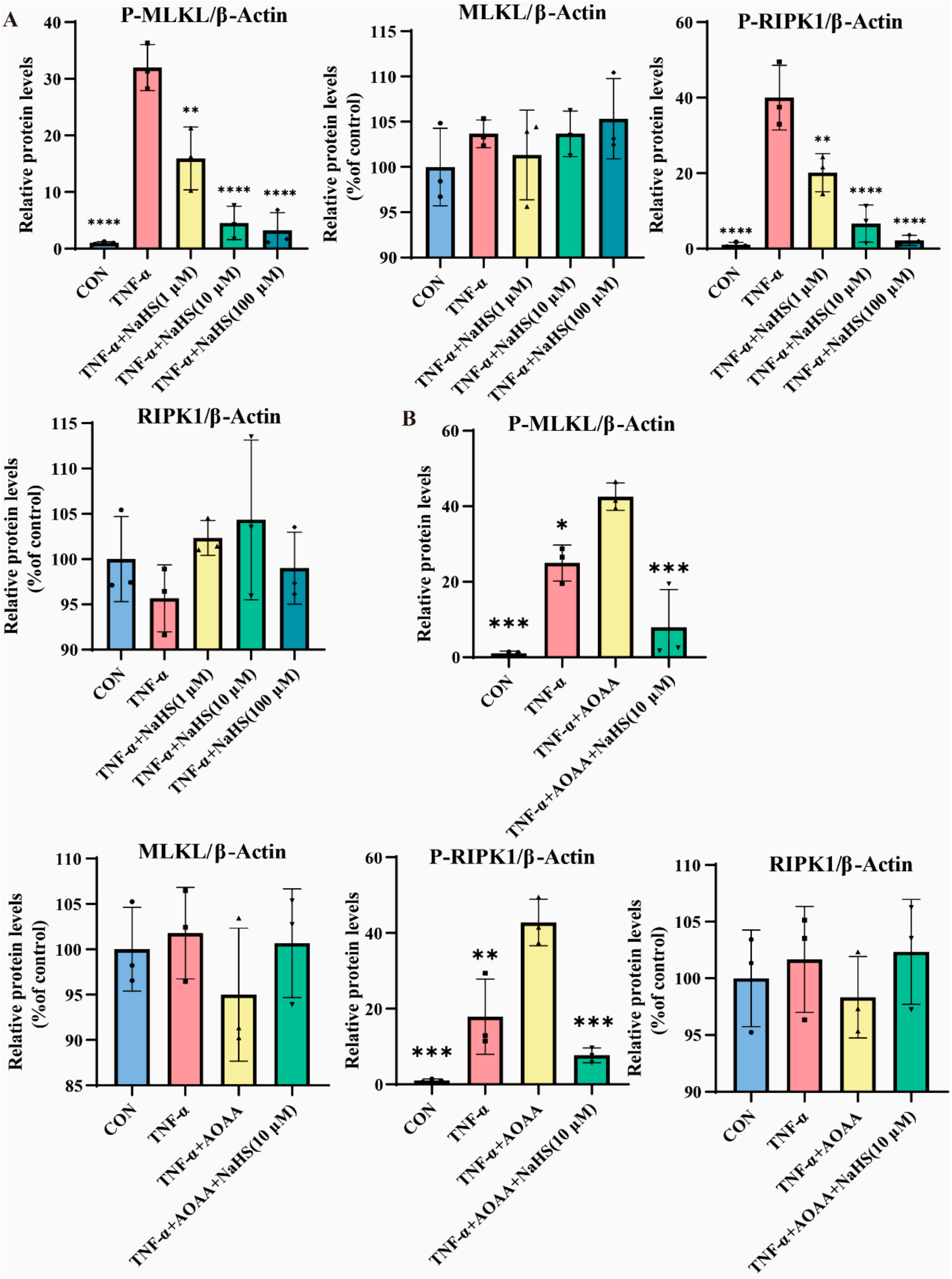

Figure Supplement 3. (A). The statistical plots of Western blot analysis for RIPK1, P-RIPK1, MLKL, P-MLKL, and β-actin in control, TNFα and TNFα+NaHS (1μM, 10μM and 100μM) groups of Y1 cells. Data are presented as mean ± standard deviation (SD). ****P < 0.0001 and **P < 0.01 indicate statistically significant differences compared to TNFα group. (n = 3 in each group) **(B).** The statistical plots of Western blot analysis for RIPK1, P-RIPK1, MLKL, P-MLKL, and β-actin in control, TNFα, TNFα+AOAA and TNFα+AOAA+NaHS (10μM) groups of Y1 cells. Data are presented as mean ± standard deviation (SD). ****P < 0.0001 and **P < 0.01 indicate statistically significant differences compared to TNFα+AOAA group. (n = 3 in each group)
